# Assessment of Hope in Pediatric Oncology: Development, Content and Face Validation of a Parental Questionnaire

**DOI:** 10.1111/hex.70388

**Published:** 2025-08-19

**Authors:** Laurine Milville, Pascal Antoine, Sophie Lelorain

**Affiliations:** ^1^ Univ. Lille, CNRS, UMR 9193 – SCALab – Sciences cognitives et Sciences Affectives Lille France; ^2^ Univ. Lausanne ‐ Institut de Psychologie, Laboratoire de psychologie de la santé, du vieillissement et du sport Lausanne Suisse

**Keywords:** content validity, development questionnaire, face validity, parental hope, pediatric cancer

## Abstract

**Introduction:**

We outline the steps in creating and evaluating the content and face validity of the Questionnaire on Parental Hope in Pediatric Oncology (Q‐PHPO), which measures the understanding of information provided by healthcare professionals (Part 1) and hope as experienced and perceived by parents (Part 2).

**Methods:**

The development of the Q‐PHPO was based on a literature review and 14 interviews with parents whose children had been diagnosed with cancer. The content and face validity were verified both quantitatively and qualitatively by assessing the clarity, relevance, and discomfort caused by the questionnaire, with input from expert parents (*n* = 7) and professionals (*n* = 10).

**Results:**

In terms of clarity, most items had an item content validity index (I‐CVI) ≥ 0.80 and a kappa (*K*) value ≥ 0.74. Despite more nuanced results being obtained for relevance, most items had an I‐CVI ≥ 0.70 and a *K* value ≥ 0.74. After revision, the first part of the Q‐PHPO includes two dimensions: the child's illness (5 items) and the child's psychosocial well‐being (4 items). The second part contains 20 items divided into three dimensions: the child's illness (6 items), the child's psychosocial well‐being (9 items), and the parent's psychosocial well‐being (5 items).

**Conclusions:**

The results provide evidence of the content and face validity of the Q‐PHPO. Further testing will confirm the questionnaire's construct and criterion validity and its reliability, making it a potentially valuable tool in pediatric oncology departments.

**Patient or Public Contribution:**

In this study, we adopted a Patient and Public Involvement and Engagement approach, actively involving patients, healthcare professionals, and researchers as key contributors to the development and evaluation of the Q‐PHPO questionnaire. Parents of children in remission from leukemia and healthcare professionals provided valuable insights based on their lived experiences, influencing the content of the questionnaire, and contributed by assessing the relevance and clarity of the questionnaire items. Researchers specializing in questionnaire development and oncology reviewed and refined the instrument, ensuring its validity and suitability.

**Abbreviations**
I‐CVI: item content validity index
K: Kappa
PPIE: Patient and Public Involvement and Engagement
Q‐PHPO: Questionnaire on Parental Hope in Pediatric Oncology
S‐CVI/Ave: scale content validity index, average method

## 1 Introduction

Assessing the parental experience using Parent Reported Outcomes is essential, both in research and clinical practice. Among the tools specifically developed for this population, the Parenting Stress Index [1] is frequently used to assess stress associated with the caregiving role [2, 3]. Recently, new questionnaires specifically developed for parents of children with cancer have emerged. These tools assess, for example, parental worries [4], resilience in the face of illness [5], or fear of cancer recurrence [6]. Yet, no validated instrument currently measures parental hope, despite its recognition as a central psychological resource—an inner strength and a key reference point for parents facing illness uncertainty [7].

Parents define hope through knowledge, belief, wish, expectation, or faith in God or any figure of a higher power [8, 9, 10, 11, 12]. In all cases, a consensus on the positive, future‐oriented aspect of hope emerges. Parents emphasize its multidimensionality, valuing its plurality [11]. Many classifications exist, including child‐centered and parent‐centered hope [13], hope related to illness or well‐being [14], and future and present‐oriented hope [8]. Various facets exist within these dimensions, such as the hope of cure, the hope that the child will always feel loved, and the hope that the child will have the best possible quality of life [13].

The importance given to these facets changes over the course of the child's illness, depending on the child's prognosis and health status [8, 12, 13]. Restructuring specific hopes seems to play an important role in parents' adjustment. By assessing personal resources, Granek et al. [8] compared the mechanisms related to hope with those related to stress in individual‐environment transactions. This was followed by increased or decreased hope (or stress) and, finally, the implementation of appropriate coping strategies.

Several studies have also investigated the adaptive nature of hope. Ninety percent of parents believe that hope is essential for coping with their child's illness [12]. Hope is valuable because it is both positive and forward‐oriented. The positive thinking encouraged by hope [7] “*keeps you alive and gives you the strength to go on living*” said a mother of a child with cancer [9] (p. 267). Looking ahead allows parents to project, plan, and prepare for the worst. Hope gives parents the strength to be realistic by enabling them to project a positive future [7, 8]. Furthermore, parents stay motivated in the face of their daily caregiving responsibilities by projecting a future in which their child can achieve his or her dreams [8].

Several health and treatment‐related factors can influence parents' hope. For example, perceiving their child as having a good quality of life despite illness can foster hope. Conversely, the child's decline, traumatic side effects, lack of symptom control, fear of relapse, and dissatisfaction with care can weaken hope [9, 12]. Changes in a child's health status can also cause shifts in the form of parental hope. For example, if the condition worsens, hope for a miracle, more time together, or relief from suffering may increase, while future‐oriented hope diminishes [8].

Parental resources also affect hope. Limited parental resources, such as a feeling of weakness, fear of facing illness, or poor social and economic status, can undermine parents' hope [9]. Conversely, managing daily life, controlling thoughts, humor, and accepting an uncertain future are, according to parents' perceptions, at the root of their hope [9, 14].

The caregiver–parent relationship also supports parental hope. A strong bond with healthcare teams reinforces both hope and trust [7, 9, 12, 15].

Measuring parental hope quantitatively remains challenging. Most studies use the Hope Scale [16], based on Snyder's theory [17], which defines hope as a two‐dimensional cognitive process. The motivational dimension (agency) is described as confidence in achieving an attainable goal, and the operational dimension is characterized by the ability to seek solutions to achieve this goal [16]. This theory was developed following general population research on the cognitive processes associated with the desire to achieve a positive goal [18]; this may explain why this theory appears to differ from the way that parents define hope. Indeed, as described above, parents perceive hope as positive, multidimensional, future‐oriented, and sometimes irrational. Furthermore, some researchers, especially those in the field of pediatric palliative care, have developed their own items to measure the different facets of the hope of parents facing their child's cancer [13, 19, 20].

We therefore consider it important to design a questionnaire that measures the specific state (not trait) hope of parents of pediatric cancer patients throughout the illness: the Questionnaire on Parental Hope in Pediatric Oncology (Q‐PHPO). This article presents the development of the questionnaire, along with its content and face validation, crucial first steps in any questionnaire's validation [21, 22]. Indeed, developing valid measures that accurately define and capture the concept of interest is a fundamental yet sometimes overlooked prerequisite for future research, which will then rely on these questionnaires to test hypotheses [22].

## 2 Methods

This study followed two main steps: (1) content determination and questionnaire development, and (2) assessment of content and face validity. The second step included three phases: quantitative expert evaluation, qualitative expert evaluation, and questionnaire revision.

### 2.1 Content Determination and Questionnaire Development

Several recommendations were followed to determine the content of and develop the questionnaire [23, 24].

First, the questionnaire aimed to assess parental hope as experienced by parents of children with cancer, aged 1 month to 18 years, regardless of cancer type or care pathway. The format consisted of statements with Likert‐scale responses.

The Q‐PHPO was developed from a literature review and 14 semi‐structured interviews with parents faced with their child's cancer^1^. The electronic databases Psych Info, Psych Articles, PubMed, Academic Search Complete, and Psychology and Behavioral Sciences Collection were searched for articles. The keywords used were as follows: hope OR hopefulness OR meaning of hope OR parental hope AND pediatric cancer OR pediatric oncology OR child with cancer OR children with cancer. The inclusion criteria were that the article be peer reviewed, published between January 2000 and December 2024, and a qualitative study. Nine articles were identified (Supporting Information S1: Tables 1 and 2).

By listing the dimensions of hope and the verbatims, we were able to identify the attitudes related to and the facets of parental hope (Supporting Information S1: Tables 1 and 2). Inductive and deductive methods were used to define the concept and identify the different dimensions that compose parental hope [24].

A working group—composed of the research investigator, a specialist in questionnaire validation, an oncopsychologist, and a pediatric oncologist—collaboratively defined the questionnaire structure and drafted the items.

### 2.2 Content and Face Validity

Once the questionnaire was developed, we verified its content and face validity. Content validity assesses whether the questionnaire adequately and completely captures the intended concept (Allen et al., 2023 [25, 26]), ensuring it is both appropriate and exhaustive [27, 28]. Face validity assesses the clarity and relevance of the questionnaire from the respondent's perspective (Allen et al., 2023). In short, content validity concerns the substance, while face validity concerns the form.

The content and face validity of the Q‐PHPO were assessed by a panel of experts specializing in the field of research studied. Some researchers recommend recruiting between 3 and 10 experts to determine content validity [25, 26, 29].

This study adopts an expanded conception of expertise, acknowledging that parents, healthcare professionals, and researchers each contribute essential and complementary knowledge. In this context, the Patient and Public Involvement and Engagement (PPIE) approach played a central role by involving patients and the public as full‐fledged participants throughout the research process.

Within this framework, three distinct groups were involved. The first group consisted of seven parents whose child was in remission from leukemia. Their lived experience makes them key contributors to the development and evaluation of the questionnaire [30, 31, 32]. The second group comprised 10 healthcare professionals with clinical expertise in pediatrics, including psychologists and pediatricians. Most specialized in pediatric oncology, while others had expertise in neuropediatrics, pediatric palliative care, or hospital home services. These two groups actively evaluated the study, providing direct input to refine the questionnaire. Lastly, the third group consisted of researchers specializing in questionnaire development and oncology. While also part of the PPIE process, their role focused on reviewing the questionnaire based on their expert knowledge [33].

Each of the expert‐parents and expert‐professionals was asked to evaluate the Q‐PHPO in two successive steps: quantitatively and qualitatively [29]. Both assessments were conducted using a rating grid emailed to the experts.

After the Q‐PHPO was reviewed, experts evaluated each item for clarity, relevance to hope, and potential discomfort via a 5‐point scale ranging from “strongly agree” to “strongly disagree” [34, 35, 36]. They could comments, suggest alternative wording and indicate missing aspects of hope.

Ease and time of completion, clarity of instructions, and discomfort caused by reading the questionnaire were also assessed.

#### 2.2.1 Phase 1: Quantitative Expert Review

The validity of the Q‐PHPO was quantitatively assessed by two expert groups: parents and professionals. The clarity (face validity) and the relevance in terms of form and content (face and content validity) were evaluated for each item in both parts of the questionnaire (Supporting File 1). First, two indices were calculated [24, 25, 26, 37]: the item‐level content validity index (I‐CVI), the proportion of experts rating the item as valid (4 or 5 on the Likert scale); and the scale content validity index/Ave (S‐CVI/Ave), the average I‐CVI for each dimension. When expert numbers exceed five, items with I‐CVI < 0.78 or S‐CVI/Ave < 0.90 should be revised or deleted [26, 36].

In addition to the CVI, Cohen's kappa (*K*) value [38] was calculated to avoid the validation of items resulting from the random agreement between experts [39]. To measure multiexpert agreement, the modified version of Fleiss was used, whose formula is *K* = $\frac{\left(\mathrm{Pa}-\mathrm{Pc}\right)}{1-\mathrm{Pc}}$, where Pa is the proportion of experts rating the item as relevant (i.e., the I‐CVI), and *P*_c_ is the probability of chance agreement. Kappa values are considered excellent, good, or fair if they are greater than 0.74, between 0.60 and 0.74 and between 0.40 and 0.59, respectively [40]. It is recommended that items with a kappa value less than 0.40 be deleted [26, 34].

#### 2.2.2 Phase 2: Qualitative Expert Review

The second step involved semi‐structured interviews with each expert. After completing the grid (Supporting File 1), experts sent it to the investigator. Their responses guided the interviews. Most expert‐parents and half the professionals preferred phone interviews; others chose videoconferencing.

The research investigator began the interview by asking the following question: “*Overall, how did you find the questionnaire?*” The research investigator then specifically asked the experts about any instructions and items that they had rated as unclear or irrelevant, along with their comments. During the interview, the investigator took detailed notes that carefully used the participants' own vocabulary. Then, by rephrasing what the participant had said, the investigator checked that he or she had understood the explanation correctly. The interviews aimed to understand each expert's views on the Q‐PHPO, discuss their feedback from the evaluation grid, and collaboratively develop ideas [35, 41]. These discussions also allowed reflection on possible revisions or deletion of certain items in consultation with the experts.

#### 2.2.3 Phase 3: Questionnaire Revision

Once all of the interviews were conducted, experts' comments were systematically processed [35]. Comparing quantitative and qualitative feedback guided item revision or deletion. Based on the literature and to reflect parents' perceptions, decisions followed:

– An I‐CVI < 0.78 from parent experts led to major revision. Independent of the professionals' assessments, if the parents assessed the item as irrelevant or unclear, it had to be revised.
– An I‐CVI < 0.78 from both groups led to major revision or deletion, informed by comments. The experts' ratings were considered a complementary element in the validation of the item.
– A *K* < 0.40 (from either group) led to deletion.
– Expert comments suggesting improvements could lead to minor revisions, even if I‐CVI > 0.78 and *K* > 0.40.

Decisions were made collectively by the investigator, a questionnaire development expert, and a psycho‐oncology expert.

## 3 Results

### 3.1 Content Determination and Questionnaire Development

The questionnaire consists of two parts.

The first part, “*What you were told*,” aimed to assess parents' perception of the optimism conveyed by the healthcare professionals how parents perceived the optimism expressed by healthcare professionals, without aiming to evaluate the parents' own level of hope. This first part consists of 10 items and two dimensions: the child's illness (6 items) and the child's psychosocial well‐being (4 items). These dimensions and items were generated based on the second part of the questionnaire.

The second part, entitled “*Deep down, your hopes, your wishes, your expectations, your feelings,*” aims to assess hope on the basis of parents' feelings and subjectivity.

First, according to the literature and interviews with parents, hope is defined as the knowledge, belief, wish, expectation, or belief in God or a spiritual being [8, 9, 10, 11, 12] (Supporting Information S1: Table 1).

Since parents linked hope to uncertainty, we excluded the definition of hope as knowledge. We also excluded the reference to faith or spirituality because it appeared in only two articles and was rarely mentioned by the French parents we interviewed. Therefore, the following definition was introduced in the second part of the questionnaire: “*You have just answered questions about what medical staff told you. Now we'd like to understand your deepest hopes, wishes, expectations and feelings*.” The different facets of hope were preceded by the heading “Deep down, *I believe in the possibility that*”. This formulation was preferred to “I *hope that*” or “*I wish that*” to avoid uniformly positive responses of “*strongly agree.*”

Second, 26 facets of hope were identified from the literature and interviews with parents (Supporting Information S1: Table 2). We excluded four facets: hope for remission (not relevant for children already in remission), hope for the end of cancer (overlapping with hope for cure and effective treatments), and hope related to faith or spirituality. Additionally, we clarified the hope that the child will live their life by focusing on three areas suitable for children and adolescents: having a fulfilling social life, participating in physical activities and sports, and playing the games they enjoy. This approach ensured precision and avoided overlap with the hope for a normal life. This second part is therefore composed of 24 items organized into four dimensions: the child's illness (6 items), the child's psychosocial well‐being (8 items), the medical staff (2 items), and the parent's psychosocial well‐being (8 items). The child illness dimension covers hopes related to health improvement, pain reduction, and the absence of short‐ and long‐term effects. The child's psychosocial well‐being dimension includes hopes for the child's overall development and quality of life across time. The medical staff dimension encompasses hopes centered on the healthcare providers' expertise and ability to effectively care for the child. The parent psychosocial well‐being dimension reflects hopes concerning the parent's emotional, relational, and personal well‐being, beyond the caregiving role. Ultimately, this classification aligns with those previously proposed in the literature, particularly the distinction between child‐centered and parent‐centered hope [13].

### 3.2 Content and Face Validity

#### 3.2.1 Phases 1 and 3: Quantitative Expert Review and Revision

##### 3.2.1.1 Relevance in Terms of Both Form and Content (Face and Content Validity) ‐ Part 1

For the expert‐parent group, the I‐CVI ranged from 0.71 to 0.86. Four items were assessed as irrelevant. Of these, two were also rated as irrelevant by the expert professionals. For the expert‐professional group, the I‐CVI ranged from 0.60 to 1 (Table 1).

**TABLE 1:** Relevance: Content analysis via the I‐CVI and S‐CVI/Ave on the basis of parents' and healthcare professionals' evaluations.

Dimension	CVI relevance					
	Parent			Pro		
	I‐CVI range	Items with I‐CVI < 0.78	S‐CVI/Ave	I‐CVI range	Items with I‐CVI < 0.78	S‐CVI/Ave
Part 1						
Child's Illness	0.71–0.86	1a*, 2a*, 3a*, 4a*	0.76	0.6–1	2a**, 4a**, 6a	0.8
Child's Psychosocial Well‐Being	0.86	—	0.86	0.8–1	—	0.9
Part 2						
Child's Illness	0.71–0.86	1b*	0.83	0.6–0.9	4b, 6b	0.81
Child's Psychosocial Well‐Being	0.86	—	0.86	0.7–0.9	12b	0.83
Medical Staff	0.71	15b*, 16b*	0.71	0.6–0.7	15b**, 16b**	0.65
Parent's Psychosocial Well‐Being	0.57–0.86	17b*, 18b*, 20b*, 21b*	0.77	0.5–0.8	17b**, 18b**, 19b, 20b**, 21b**, 22b, 24b	0.61

* Items with CVI values < 0.76 scored by parents: Major revision.

** Items with CVI values < 0.76 scored by parents and professionals: Major revision or deletion; —: No items with I‐CVI < 0.78.

The S‐CVI/Ave values for the child illness dimension and child psychosocial well‐being dimension were 0.78 and 0.86, respectively, for the expert‐parent group and 0.80 and 0.90, respectively, for the expert‐professional group (Table 1).

For the expert‐parent group, the *K* value ranged from 0.65 to 0.85. Six items were rated as excellent, and 4 items were rated as good. For the expert‐professional group, the *K* value varied between 0.50 and 1. Seven items were rated as excellent, 2 items were rated as good, and 1 item was rated as fair (Table 3).

*Revision* (based on the criteria described in phase 3 of the Methods section)

Items 1a and 3a required major revision.

Items 2a and 4a required major revision or deletion (Supporting Information S1: Table 3).

##### 3.2.1.2 Relevance in Terms of Both Form and Content (Face and Content Validity) ‐ Part 2

For the expert‐parent group, the I‐CVI ranged from 0.57 to 0.86. Seven items were assessed as irrelevant. Of these, six were also rated as irrelevant by the expert professionals. For the expert‐professional group, the I‐CVI ranged from 0.50 to 0.90 (Table 1).

The S‐CVI/Ave values for the dimensions of child illness and child psychosocial well‐being were 0.78 and 0.86, respectively, for the expert‐parent group and 0.80 and 0.90, respectively, for the expert‐professional group (Table 1).

For the expert‐parent group, the *K* value varied from 0.40 to 0.85. Seventeen items were considered excellent, 6 items were considered good, and 1 item was considered fair. For the expert‐professional group, the *K* value ranged from 0.35 to 0.90. Fourteen items were rated as excellent, 1 item was rated as good, 7 items were rated as fair, and 2 items were rated as poor (Table 3).

*Revision*

Item 1b required major revision.

Items 15b, 16b, 17b, and 20b required major revision or deletion.

Items 18b and 21b were deleted (Supporting Information S1: Table 3).

###### 3.2.1.2.1 Clarity (Face Validity) ‐ Part 1

The I‐CVIs ranged from 0.86 to 0.1 for the expert‐parent group and from 0.70 to 1 for the expert‐professional group. No item was assessed as unclear by the expert parents (Table 2).

The S‐CVI/Ave values for the dimensions of child illness and child psychosocial well‐being were 0.95 and 0.93, respectively, for the expert‐parent group and 0.82 and 0.90, respectively, for the expert‐professional group (Table 2).

For the expert‐parent group, the *K* value ranged from 0.85 to 1. All items were rated as excellent. For the expert‐professional group, the *K* value ranged from 0.66 to 0.90. Eight items were rated as excellent, and 2 items were rated as good (Table 3).

*Revision*

No items required revision (Supporting Table 3).

###### 3.2.1.2.2 Clarity (Face Validity) ‐ Part 2

The I‐CVIs for the expert‐parent group and the expert‐professional group ranged from 0.71 to 0.1. One item was assessed as unclear by the expert parents (Table 2).

**TABLE 2:** Clarity: Content analysis via the I‐CVI and S‐CVI/Ave on the basis of parents' and healthcare professionals' evaluations.

Dimension	CVI clarity					
	Parents			Professionals		
	I‐CVI range	Items with I‐CVI < 0.78	S‐CVI/Ave	I‐CVI range	Items with I‐CVI < 0.78	S‐CVI/Ave
Part 1						
Child's Illness	0.86–1	—	0.95	0.7–0.9	2a, 5a	0.82
Child's Psychosocial Well‐Being	0.86–1	—	0.93	0.8–1	—	0.9
Part 2						
Child's Illness	0.86–1	—	0.98	0.7–1	4b	0.9
Child's Psychosocial Well‐Being	0.86–1	—	0.89	0.8–1	—	0.93
Medical Staff	0.71–0.86	16b*	0.79	0.9	—	0.9
Parent's Psychosocial Well‐Being	0.86–1	—	0.98	0.8–1	—	0.89

* Items with CVI values < 0.76 scored by parents: Major revision; **Items with CVI values < 0.76 scored by parents and professionals: Major revision or deletion; —: No items with I‐CVI < 0.78.

The S‐CVI/Ave values for the child's illness, child's psychosocial well‐being, medical staff and parent's psychosocial well‐being dimensions were 0.98, 0.89, 0.77, and 0.98, respectively, for the expert‐parent group and 0.90, 0.93, 0.90, and 0.89, respectively, for the expert‐professional group (Table 2).

The K value varied between 0.85 and 1 for the expert‐parent group and between 0.66 and 1 for the expert‐professional group (Supporting Information S1: Table 3). Twenty‐three items were rated as excellent, and 1 item was rated as good by the expert parents and expert professionals (Table 3).

**TABLE 3:** Number of items and items in each kappa category of relevance and clarity in on the basis of parents' and healthcare professionals' evaluations.

Dimension	Kappa relevance							Kappa clarity			
	Parents			Professionals				Parents		Professionals	
	Excellent	Good	Fair	Excellent	Good	Fair	Poor/Item	Excellent	Good	Excellent	Good
Part 1											
Child's Illness (6)	2	4	—	3	2	1	—	6	—	4	2
Child's Psychosocial Well‐Being (4)	4	—	—	4	—	—	—	4	—	4	—
Part 2											
Child's Illness (6)	5	1	—	5	—	1	—	6	—	5	1
Child's Psychosocial Well‐Being (8)	8	—	—	8	—	—	—	8	—	8	—
Medical Staff (2)	—	2	—	—	—	2	—	1	1	1	—
Parent's Psychosocial Well‐Being (8)	4	3	1	1	1	4	**2/** ***18b*** ***,21b***	8	—	8	—

*** Item with low kappa values: Deletion; —: No items

*Revision*

Item 16b required major revision (Supporting Information S1: Table 3).

#### 3.2.2 Phase 2: Qualitative Expert Review and Revision

Overall, both expert parents and expert professionals found the Q‐PHPO to be easy to complete, to require a reasonable amount of time, and to be aligned with the experiences of parents dealing with their child's cancer diagnosis.

Despite this positive assessment of the Q‐PHPO, some revisions were made.

First, three items (1a, 3a, and 1b) needed major revisions based on quantitative analyses. Experts noted that the instructions were unclear about timing, since hope can change during treatment. Therefore, the instructions were updated to tell parents to refer to what physicians recently communicated for Part 1, and to their current feelings for Part 2 (Table 4).

**TABLE 4:** Minor and major revisions and deletions.

Assessment	Item initial	Comment	Action	Revision
Minor revision	5a Sequelae 5b. Grows up without sequelae	Lacks precision on the type of sequelae and overlaps with the pain management item if referring to short‐term sequelae	Revision	5a. Long‐term psychological, physical, or cognitive sequelae 5b. Grows up without psychological, physical, or cognitive sequelae
	7a. Normal school education 9b. Following a normal school education	Item unsuitable for children in special or alternative education and does not apply to toddlers	Revision	7a. School education 9b. Fulfilling school experience Added the option “Not applicable”
	3b. Receiving effective treatment	Item inappropriate once treatment has begun	Revision	3b. Has received or is receiving effective treatments
	4b. Not physically suffering or no longer physically suffering	Item imprecise about the causes of suffering (illness or iatrogenic) and inappropriate for assessing discomfort	Revision	4b. Has less or no more physical suffering or discomfort due to the illness or treatments (e.g., side effects)
Major revision*	1a. The cure	Instructions unclear due to the fluctuation of hope during the treatment process	Revision	[Part 1] We will ask you to recall what you have recently been told by the medical staff about your child's current and future health. [Part 2] Now, we would like to understand, at this moment, what your deep hopes, wishes, expectations, and feelings are.
	3a. Effectiveness of treatment			
	1b. Being cured			
Major revision or Deletions**	2a. Life expectancy	Item rarely mentioned by healthcare professionals and overlaps with the cure item	Deletion	
	4a. Pain management	Item imprecise about the causes of suffering (disease or iatrogenic) and excludes discomfort	Revision	4b. The management of physical suffering or discomfort experienced by the child due to the illness or treatments (e.g., side effects).
	15b. My child will be cared for by physicians who really understand his or her illness.	Items assess parents' confidence in the medical staff	Deletion	
	16b. The medical staff is competent to care for my child.		Deletion	
	17b. My child will receive as much love as possible.	Items assess the parent's ability to cope with the situation and manage their emotions.	Deletion	
	20b. Finding meaning in this situation		Deletion	
Deletions***	18b. Making my child happy		Deletion	
	21b. Gain some peace of mind		Deletion	

The results of the quantitative analyses indicated that 6 items needed to be revised or deleted to ensure the content validity of the questionnaire. Items 15b, 16b, 17b, and 20b were deleted because most experts thought they measured concepts other than parental hope. For example, Item 15b (“*My child should be cared for by physicians who really know his or her illness*”) was used to assess parents' trust in medical staff. Similarly, Item 17b (“*Give my child as much love as possible*”) reflected parents' ability to manage their situation. Item 2a (“*Life expectancy*”) was also deleted most professionals rarely mentioned life expectancy to parents, and it overlapped with Item 1a ("*hope for cure*"). Item 4a, (“*Pain management*”) was revised to include the causes of pain (illness or iatrogenic) and the experience of discomfort (Table 4).

Next, after the qualitative assessment, we made minor revisions to six items by clarifying their formulations to ensure the face validity of the questionnaire (5a, 5b, 7a, 9a, 3b, and 4b). For example, the item “*after‐effects*” was clarified by revising it to “*long‐term psychological, physical, or cognitive after‐effects.*” Another minor change was adding a “*Does not apply*” option for items about schooling (7a and 9b), to accommodate parents of very young children (Table 4).

The qualitative analysis revealed some missing items, thereby limiting the content validity of the questionnaire. An item concerning the well‐being of siblings and another item concerning the child's ability to cope were added (Table 5) despite the fact that these facets of hope were not identified in the existing literature or in the interviews initially used to develop the questionnaire. However, they were spontaneously mentioned by several parents during the validation phase and were considered relevant to capturing the lived experience of parental hope.

**TABLE 5:** Revision: Missing items and instructions.

Type of modification	Comment	Revision (additional element)
Missing items	Added an item on the well‐being of siblings	The sibling(s) of my ill child can be fulfilled.
	Added an item on the child's ability to cope with the situation	My child can overcome this situation.
Instruction	Added a sentence to legitimize the discomfort parents may feel when reading the items, particularly the items on a cure, life expectancy, and the effectiveness of treatments.	Some of the questions may make you feel uncomfortable, and you may need to take some time to think about them. Remember that there are no right or wrong answers ‐ your immediate feelings are probably a good indication of how you feel.
	Expanded the instructions by describing the Likert scale	[Part 1] Using the scale below, please indicate the extent to which each of the following statements is very pessimistic (1) or very optimistic (5). You can also indicate that medical staff did not give you any information by ticking “I was not told anything”. [Part 2] Using the scale below, please indicate the extent to which you strongly disagree (1) or strongly agree (5) with each of the following statements. You can also indicate that the item does not apply to your situation by ticking “Does not apply”.

In addition, reading the Q‐PHPO, especially the items concerning a cure, life expectancy, and treatment effectiveness, could be uncomfortable for some parents and professionals. On their recommendations, we added a sentence to the instructions to legitimize this discomfort (Table 4). The instructions also now clearly explain the Likert scale for both parts of the questionnaire (Table 5). It is also worth noting that all parents and healthcare professionals recognized the distinction between the optimism conveyed in medical information, assessed in the first part of the questionnaire, and hope, which may differ from the medical information provided, assessed in the second part of the questionnaire.

## 4 Discussion

This study describes the process of developing and validating the content and appearance of the Q‐PHPO, which aims to measure the hope of parents faced with their child's cancer.

The first step involved defining its content. Based on a literature review and parent interviews, the questionnaire was structured into two parts.

The first part aims to assess the medical information provided by healthcare professionals and understood by parents. This is relevant, as a child's condition affects parental hope. Parental hope can be threatened by a lack of control over symptoms, uncomfortable care, fear of relapse, or uncertainty about diagnosis and treatment [9, 12]. Conversely, clear, optimistic communication can foster hope [7, 12]. Since care context shapes hope, it is helpful to assess parents' understanding of the illness as a control variable in the second part.

In the second part, parents are encouraged to refer to their wishes, expectations, and feelings. Hope is then introduced with the statement “Deep down, *I believe in the possibility that.*” This definition, which is based on the oncopediatric literature, is also consistent with the perception of the general population: “*the belief that a future outcome will be positive, combined with a desire for that outcome*” [42]. Thus, the Q‐PHPO distinguishes optimism (part 1) from hope (part 2)—an important distinction, as hope may persist even when expectations are not realistic [43]. Therefore, the Q‐PHPO captures a complex, subjective experience rooted in parental reality, rather than mere probability.

Next, Expert‐parent and expert‐professional ratings assessed content and face validity. Clarity ratings were generally positive, while relevance ratings were more variable, prompting revisions. Some items were deleted, including two about medical staff, as they reflected trust more than hope [44, 45]. Four items from the parents' psychosocial well‐being dimension were also deleted because, according to the experts, they assessed parents' perceptions of their ability to cope with the situation and their emotions; this reflects the concept of self‐efficacy [46]. Additionally, Zhou and Kam highlighted that self‐efficacy is conceptually similar to hope, as defined by Snyder [47]. The deletion of these items allowed both the avoidance of any similarity to the Hope Scale [16] and the full achievement of the aim of this study: the development of a questionnaire that is conceptually close to parents' perceptions of hope.

In addition, experts also suggested adding an item about siblings. The literature highlights the fact that siblings have disrupted family experiences and therefore experience neglect, insecurity and negative emotions such as isolation, anxiety and sadness. Despite these difficulties, siblings play a crucial role in maintaining family unity by adopting a positive attitude and supporting both their ill siblings and their parents [48].

The first part of the Q‐PHPO comprises nine items: 5 items for the child's illness dimension and 4 items for the child's psychosocial well‐being dimension. The second part of the questionnaire contains 20 items organized into three dimensions: 6 items for the child's illness dimension, 9 items for the child's psychosocial well‐being dimension and 5 items for the parent's psychosocial well‐being dimension (Supporting Information S1: Files 2 and 3).

## 5 Strengths and Limitations

This study has several notable strengths.

First, the development of a questionnaire to measure parents' hope for their child's cancer is innovative. No existing questionnaire measures the extent to which parents have specific hope regarding the disease, their child's psychosocial well‐being, and their own psychosocial well‐being.

Second, the co‐construction of the questionnaire in partnership with expert‐parents and expert‐professionals helped ensure its relevance and robustness [30]. Moreover, the complementary perspectives of these expert groups helped validate this coherent and relevant questionnaire for both healthcare professionals and parents, facilitating its adoption and use.

Item generation based on quantitative and qualitative analysis strengthened the reliability and robustness of questionnaire validation [29]

Although the interviews were informative, the lack of cognitive interviews is a limitation.

Cognitive interviews, which involve observing participants' thoughts while answering, could provide further insight into how items are understood [41] and should be a next validation step.

The protocol was not iterative with multiple cycles until saturation—a more time‐intensive but potentially stronger approach [34]. Recruitment was limited by participant availability. Nevertheless, interviews were rigorous, with participants invited to suggest and discuss changes.

The questionnaire was developed in French, limiting its use to French‐speaking populations and requiring translation for wider use. It is not suitable for all parents, especially those with children in palliative care. The Q‐PHPO reflects experiences of parents of children in treatment or remission, focusing on hope for improvement, which may not apply to end‐of‐life situations.

Although healthcare professionals worked with children with various cancers, only parents of children with leukemia participated. This limits generalizability to other cancer types, such as solid tumors, including brain tumors. Future validation should include parents of children with other cancers to broaden applicability.

Moreover, further tests are needed to confirm the questionnaire's validity, including construct validity, criterion validity, and reliability [21, 23]. These steps are essential to strengthen its psychometric properties and ensure robustness across clinical settings.

## 6 Conclusion

The aim of the present study was to develop and verify the content and face validity of the Q‐PHPO, a questionnaire aimed at measuring the hope of parents faced with their child's cancer in a French‐speaking European context. The results provide evidence of the questionnaire's content and face validity. The revisions made based on the recommendations of parent and professional experts enabled us to improve the questionnaire and propose a satisfactory version of the Q‐PHPO.

## Research and Clinical Implications

The use of the Q‐PHPO in research will elucidate the links between hope status, as perceived and felt by parents, and other variables related to parents' experiences.

Clinically, the questionnaire could be a useful tool in oncopediatric departments. The results of this questionnaire can be considered “patient‐reported outcomes” (PROs) and, more specifically, “parent‐reported outcomes.” The clinical use of PROs is known to significantly improve the care of patients in the field of adult oncology, particularly by improving communication between healthcare professionals and patients [49].

## Author Contributions

**Laurine Milville:** conceptualisation, methodology, formal analysis, investigation, writing – original draft, writing – review and editing. **Sophie Lelorain:** conceptualisation, methodology, formal analysis, writing – original draft, supervision, funding acquisition. **Pascal Antoine:** conceptualisation, methodology, formal analysis, writing – original draft, supervision.

## Ethics Statement

This study was approved by the University of Lille. The research coordinator ensured that the participants consented to participate in this study and that they were informed of the purpose of the research, confidentiality and their right to terminate their participation at any time.

## Conflicts of Interest

The authors declare no conflicts of interest.

## Supporting information

Final_Appendices.

## Data Availability

The data that support the findings of this study are available on request from the corresponding author. The data are not publicly available due to privacy or ethical restrictions.

## Endnotes

1 These interviews were conducted as part of a published study aimed at understanding the nature of hope in couples whose child has cancer (doi: 10.1016/j.ejon.2024.102690). On reasonable request, some of the interviews, which are useful in understanding parents’ definitions of hope, can be made available to readers.
