# Supplementary material for: Assessment of Hope in Pediatric Oncology: Development, Content and Face Validation of a Parental Questionnaire
Source: Health Expect. 2025 Aug 19;28(4):e70388. doi: 10.1111/hex.70388 (PMC12363407; doi:10.1111/hex.70388)
Supplement: Supplementary file 1 — Final_Appendices. [file HEX-28-e70388-s001.docx]

**Supplementary table 1**

*Definition of hope: quotation and reference*

| **Definition** | **Quotation** | **Reference** | **Interviews** |
| --- | --- | --- | --- |
| **Future** | Hope is focused on something in the future. | Kylmä et Juvakka .2007 | - |
| **Positive** | Hope is connected to being positive, and, I believe hope and being positive are healers in a way. | Barrera et al., 2013 | - |
|  | What hope means to me is. you know, is waiting and it’s a positive feeling for a positive future. or something that you’re looking forward to | Granek et al., 2013 | - |
|  | It (hope) is a very positive word | Kylmä et Juvakka .2007 | - |
|  | Hope is also about seeing life in a positive light | - | Interviews |
| **Knowledge (certain)** | Hope is knowing that even in the midst of the absolute worse that everything will still be okay | Conway et al.,. 2017 | - |
|  | Willing things to go a certain way | Conway et al., 2017 | - |
|  | I don’t use the word hope; I try to think positively . . . I don’t have to hope because I just know she is going to be okay. | Conway et al., 2017 | - |
|  | Knowing that the doctor is a top specialist and we received the best care possible.’ | Kylmä et Juvakka .2007 | - |
|  | I think in fact hope would be a medical certainty perhaps but I don't think that's possible. |  | Interviews |
|  | The hope I still have is to know that there is a third line of treatment | - | Interviews |
|  | The place of hope is in the willingness to say it has to happen; it has to get better. | - | Interviews |
| **Belief** | I hope that somewhere while he’s going through treatment. they do find a cure, but I know the reality of that is pretty slim. | Kamihara et al., 2015 | - |
|  | I'm hoping for a cure while he’s going through treatment, but I know that’s kind of a long shot. | Kamihara et al., 2015 | - |
|  | I am hoping that this is going to work but I am feeling in my heart that it is not. | Kamihara et al., 2015 | - |
|  | That is the ultimate, I believe she is cured...that is my ultimate hope | Granek et al., 2013 | - |
|  | I’m hoping that things will continue to be good. I believe in miracles, but as far as expecting? I don’t. I hope.’ | Granek et al., 2013 | - |
|  | Belief that my child will finally get well' | Pastistea et al., 2000 | - |
|  | It was 20% and why not us? I think that's believing | - | Interviews |
|  | Moving from tragedy to the possibility of a cure | - | Interviews |
|  | So yeah. I say to myself that there's hope in everything anyway and then if you don't believe in it then there's no hope | - | Interviews |
|  | Yes yes. the hope. we believe anyway | - | Interviews |
|  | I was losing hope. he was shaking me up and telling me. no. you’ve got to believe | - | Interviews |
| **Wish** | It’s sort of wishing for something to go the way you want it to be | Conway et al., 2017 | - |
|  | To me hope means a desire to live. | Kylmä et Juvakka .2007 | - |
|  | I wish that the life would be good for my daughter. | Kylmä et Juvakka .2007 | - |
|  | Second. we hope to hear back about the results of the bone marrow aspiration. We want to hear that they could not detect any neuroblastoma cells. I don’t know how realistic that is but I am hoping for it anyway… | McGeehin Heilferty et al., 2018 | - |
| **God or Spirituality** | Hope looks like faith and believing in God. | Conway et al., 2017 | - |
|  | In our family it is self-evident that there is no other hope than trust on God. | Kylmä et Juvakka .2007 | - |
| **Expectation** | What hope means to me is, you know, is waiting and it’s a positive feeling for a positive future, or something that you’re looking forward to | Granek et al., 2013 | - |

**Supplementary table 2**

*Facets of hope: dimension. quotation and reference*

| **Dimension** | **Facet** | **Quotation** | **Reference** | **Interviews** |  |
| --- | --- | --- | --- | --- | --- |
| **Child’s illness** | Cure | The hope is that she’s going to be one of the 30% that make it (…) my hope is that we caught it early on. | Berrera et al., 2013 | - |  |
|  |  | My hope is that she is healed. You know? Cured. | Granek et al., 2013 | - |  |
|  |  | She ‘Hope[d] of having a full recovery.’ | Granek et al., 2013 | - |  |
|  |  | I am hoping that he is going to get cured. | Kamihara et al., 2015 | - |  |
|  |  | I hope that it is going to be cured. | Kamihara et al., 2015 | - |  |
|  |  | I'm hoping for a cure while he’s going through treatment. but I know that’s kind of a long shot. | Kamihara et al., 2015 | - |  |
|  |  | First and foremost was a cure (...). But I think his hope was based somewhere on other things. even if the overall hope was, was a cure. | - | Interviews |  |
|  |  | Hope of cure | - | Interviews |  |
|  |  | Moving from tragedy to the possibility of a cure | - | Interviews |  |
|  |  | The hope that it wouldn't come back for the time being. but at the time we had hope that it would cure. | - | Interviews |  |
|  |  | I hoped that my daughter would get better. | - | Interviews |  |
|  |  | Hope is mainly for the cure | - | Interviews |  |
|  |  | I hoped that my child would be cured. | Van der Geest et al., 2015 | - |  |
|  | End of cancer | I am always hoping that she survives this and I am wrong and everyone is wrong and this thing will go away. | Kamihara et al., 2015 | - |  |
|  |  | You hope that your child is going to be. um. healthy. | Kamihara et al., 2015 | - |  |
|  |  | Belief that my child will finally get well | Patistea et al., 2000 | - |  |
|  |  | The hope that... my daughter will never have anything again | - | Interviews |  |
|  | Remission  Long life / adulthood | Hope of remission | Granek et al., 2013 | - |  |
|  |  | But my hope is that he will live to adulthood. He will grow up | Kamihara et al., 2015 | - |  |
|  |  | I just hope that he can live longer. | Kamihara et al., 2015 | - |  |
|  |  | [I hope] just for as much time as possible, and to have him as healthy as possible for as long as possible. | Kamihara et al., 2015 | - |  |
|  |  | I hope that he lives a long life [laughs] | Kamihara et al., 2015 | - |  |
|  |  | I want to see him live long enough to grow up and become an adult. | Kamihara et al., 2015 | - |  |
|  | Effectiveness of treatments | We had an 80% reduction in the tumour when they did an evaluation after chemotherapy. So, I’m hoping that the last 20% over a 40-day period almost and 2 chemo treatments got it eliminated. So. I’m hoping for a 100% reduction in the tumour. | Berrera et al., 2013 | - |  |
|  |  | The hope is to lengthen that honeymoon period; the term they use for the radiation treatment can slow down the tumour growth and potentially stop it. | Granek et al., 2013 | - |  |
|  |  | Think just hopeful that, you know, the treatment will be effective. | Kamihara et al., 2015 | - |  |
|  |  | I am hoping that this is going to work but I am feeling in my heart that it is not. | Kamihara et al., 2015 | - |  |
|  |  | [I hope] that the disease stabilizes and she has, you know | Kamihara et al., 2015 | - |  |
|  |  | To find something to slow it down and buy us some time. | Kamihara et al., 2015 | - |  |
|  |  | Today we hope for a couple of things, one, that the results of the stem cell collection are good. Third. we are hoping to have her new catheter removed. . . | - | Interviews |  |
|  |  | I hope that the results will prove me wrong | - | Interviews |  |
|  |  | The hope I still have is to know that there is a third line of treatment | - | Interviews |  |
|  | Suffering / pain | The least pain and, you know, the least suffering through that. | Kamihara et al., 2015 | - |  |
|  |  | that ‘(the) first thing is just hoping that we can get her feeling better. She can’t even walk. So, I want her to be pain free.’ | Granek et al., 2013 | - |  |
| **Child’s psychosocial well-being** |  | I want him to feel as comfortable as possible. | Kamihara et al., 2015 | - |  |
|  |  | The least pain and, you know, the least suffering through that. | Kamihara et al., 2015 | - |  |
|  |  | The hope that he won't have to live, or relive what is to come, all the suffering that E. has gone through. | - | Interviews |  |
|  |  | I hoped for a death without pain. | Van der Geest et al., 2015 | - |  |
|  | Long-term side effects | ‘[I’m hoping] just that he doesn’t get any complications, .. I wanted [him] not to get really. really sick [from treatment] | Granek et al., 2013 | - |  |
|  |  | ‘[I’m hoping] there’ll be little or minimal complications.’ | Granek et al., 2013 | - |  |
|  |  | I want to get him through this treatment as easy as possible. | Kylmä et Juvakka. 2007 | - |  |
|  |  | The hope that one day he'll be able to live with it and not be... too affected by the treatments he's had to undergo. | - | Interviews |  |
|  |  | The hope that it will grow (size) normally | - | Interviews |  |
|  | Fertility | This mother spoke again about hoping for minimal complications in desiring ‘no long-term side effects and I would really, really, like for her sterility to be intact. . .. I don’t want her sterile. I want her fertility to be intact.’ | Granek et al., 2013 | - |  |
|  | Happy | The only hope is that you just live day to day and hold off that change. You just do your best. You pray you wake up and she jumps in your bed and she’s smiling again. And that’s it. | Granek et al., 2013 | - |  |
|  |  | I am hoping that he is going to live a full life. | Kamihara et al., 2015 | - |  |
|  |  | And. um. have a happy life | Kamihara et al., 2015 | - |  |
|  |  | [I hope he can] live many years happily and as healthy as possible, without having too many effects on the quality of life. | Kamihara et al., 2015 | - |  |
|  |  | [I hope] just that it gets better and stops giving him problems so that he can enjoy his life. | Kamihara et al., 2015 | - |  |
|  |  | I want her to be happy and healthy and enjoy her life and whatever she wants to do with it. | Kamihara et al., 2015 | - |  |
|  |  | The hope that he will be happy | - | Interviews |  |
|  | Normal life | Just a long, happy, not special life, but just a regular kid. | Kamihara et al., 2015 | - |  |
|  |  | [I hope he can have] a normal life. | Kamihara et al., 2015 | - |  |
|  |  | Right now, I just want her to be healthy like a normal kid, be a human being, you know, and do all the thing[s], you know, that a kid do[es]. We want her to be healthy and act like normal children. | Kamihara et al., 2015 | - |  |
|  |  | [I hope that] he can live a normal life like a normal [age] year-old. | Kamihara et al., 2015 | - |  |
|  |  | [I hope] that the disease stabilizes and she has, you know, a normal life like everyone else with the least impact on her. | Kamihara et al., 2015 | - |  |
|  |  | [I hope my child can] make it through this stuff as whole as possible and have a normal life. | Kamihara et al., 2015 | - |  |
|  |  | Hope for the normal life | Kylmä et Juvakka. 2007 | - |  |
|  |  | Hope, too, for a more normal life for her | - | Interviews |  |
|  |  | Hope for a normal life | - | Interviews |  |
|  |  | There’s hope of having a normal life (…) with the she won’t be doing combat sports but she’s not interested in that so.. | - | Interviews |  |
|  | Schooling | I am hoping to see him graduate from high school and get married and someday have children. I mean. that would be the ultimate. | Kamihara et al., 2015 | - |  |
|  |  | For her future life as a woman. there's nothing to worry about and yeah. so there's plenty of hope there | - | Interviews |  |
|  | Childhood | We hoped he would have a more or less normal life. So what we did was go to the playroom, get on a bike and ride around the hospital | - | Interviews |  |
|  |  | The hope that he would live normally meant that he had to readapt to a lot of things anyway – his bedroom. no more carpets, not too many toys in the house. | - | Interviews |  |
|  |  | Hope by living as normally as possible (…) I asked the doctor. I said “he has to see grandpa. his granny, some friends”. | - | Interviews |  |
|  |  | Hope that maybe one day we can return to a normal life | - | Interviews |  |
|  | Job | I hope she has a good career. | Kamihara et al., 2015 |  |  |
|  |  | My little boy has transformed into a young man. I hope he can take off with his sports dream! | Wong et al., 2024 |  |  |
|  | Starting a family / getting married | Her hope of becoming a midwife | - | Interviews |  |
|  |  | My hope for him (is) that he’s going have girlfriends, get married, hopefully have children. . .. We’re hoping that this is just a blip on the radar. | Granek et al., 2013 |  |  |
|  |  | He will marry somebody that he loves. They will one way or another have children. | Kamihara et al., 2015 | - |  |
|  |  | I hope that someday I will be a granny.’ | Kylmä et Juvakka. 2007 | - |  |
| **Medicine** | Hospital expertise | We hope that this hospital will help [child] live longer a little bit you know. That is the reason we came in here for. | Kamihara et al., 2015 | - |  |
|  | Physician’s expertise | Knowing that the doctor is a top specialist and we received the best care possible | Kylmä et Juvakka. 2007 | - |  |
|  |  | To hope to “having faith in” the oncologist. | - | Interviews |  |
|  |  | I just trust him” (D/M7) or “you have to sort of really sort of put your faith into them and just trust” | - | Interviews |  |
|  | Finding a treatment | I hope that they can find a cure for this cancer. I hope that they continue doing research | Kamihara et al., 2015 | - |  |
|  |  | I hope that somewhere while he’s going through treatment. they do find a cure, but I know the reality of that is pretty slim. | Kamihara et al., 2015 | - |  |
| **Parent’s psychosocial well-being** | Making a child happy | My aim is to make him happy every day | Kamihara et al., 2015 | - |  |
|  | Overcoming the situation | We’re hoping that this is just a blip on the radar. . .. That this is just a period in his life that we’re going to get through, that we’re going to get over and that we’re going to move on. | Granek et al., 2013 | - |  |
|  |  | The hope of getting rid of this situation | - | Interviews |  |
|  |  | My goal is to get my children out of this mess | - | Interviews |  |
|  | Finding meaning | I do not have any other hope: I accept everything. and I believe that there is some reason behind all this. there is a purpose in why these difficulties have been given to me. | Kylmä et Juvakka. 2007 |  |  |
|  | Peace of mind | Hope for the future. hope for serenity, maybe not even peace, but a moment of clarity, a moment when you can breathe a sigh of relief. | - | Interviews |  |
|  |  | After that. you're often a bit upset, or you stay in a corner, you keep quiet and say nothing, and you hope it'll pass quickly and that's it | - | Interviews |  |
|  |  | We hope to be at peace | - | Interviews |  |
|  |  | The hope that you'll be able to relax... I like to say that I'd like (...) to be bored. | - | Interviews |  |
|  |  | I hope we’ll be able to get away from all these question | - | Interviews |  |
|  | Quality time. as a family | Hope is everything that we have together and the hope for being together. | Kylmä et Juvakka. 2007 | - |  |
|  |  | We hope for the life together. | Kylmä et Juvakka. 2007 | - |  |
|  |  | I hoped for a meaningful time with my child. | Van der Geest et al., 2015 | - |  |
|  |  | It's the hope that nothing bad will ever happen to us (family) again. | - | Interviews |  |
|  |  | One hope I have is to be at 5 | - | Interviews |  |
|  | Quality time. as a couple | I hope we can rescue those things we couldn't do during the treatment, enjoy the good side of the relationship, intimacy, leisure activities. and spend more time together | Silva-Rodrigues et al., 2016 | - |  |
|  |  | When my child revivers her health and can-do things by herself, hopefully we will have time for rebuilding our relationship | Yi et al., 2021 | - |  |
|  | Sympathy / Support | Probably the hope was in the sympathy received from other people | Kylmä et Juvakka. 2007 | - |  |
|  |  | There’s hope for support | - | Interviews |  |
|  |  | ‘‘I wish that the life would be good for my daughter.’ | Kylmä et Juvakka. 2007 | - |  |
|  | Sharing positives / Be a support | After recovery, we also joined a children's choir hoping to spread more positive energy to those on the same path. | Wong et al., 2024 |  |  |
| **Spirituality** | Miracle | I’m hoping that things will continue to be good. I believe in miracles, but as far as expecting? I don’t I hope.’ | Granek et al., 2013 | - |  |
|  |  | Maybe there’s a miracle, maybe things will be, you know, like I say you gotta’ hope. If you don’t hope for something it’s just not worthwhile | Granek et al., 2013 | - |  |
|  |  | I hope. you know, that he can be a miracle child. | Kamihara et al., 2015 | - |  |
|  |  | The hope that a lucky star will shine and make things change for the better. | - | Interviews |  |
|  | God | In the moments when I feel weak, like I can’t continue, my hope is almost crushed, and the only hope that carries me is the hope that God exists. | Berrera et al., 2013 | - |  |
|  |  | Hope looks like faith and believing in God. | Berrera et al., 2013 | - |  |
|  |  | Of course, I hope that my daughter will have her faith in God also in the future | Kylmä et Juvakka. 2007 | - |  |

**Supplementary table 3**

*CVI, Kappa, and evaluation of relevance and clarity for each item*

| Part 1 | Item | Relevance | | | | | Clarity | | | | |
| --- | --- | --- | --- | --- | --- | --- | --- | --- | --- | --- | --- |
|  |  | CVI | | Kappa | | Evaluation | CVI | | Kappa | | Evaluation |
|  |  | Parent | Pro | Parent | Pro |  | Parent | Pro | Parent | Pro |  |
| Child’s illness | 1a. | 0.71 | 0.9 | 0.65 | 0.9 | Revision | 1 | 0.9 | 1 | 0.9 | Valid |
|  | 2a. | 0.71 | 0.6 | 0.65 | 0.5 | Major revision or Deletion | 1 | 0.7 | 1 | 0.66 | Valid |
|  | 3a. | 0.71 | 1 | 0.65 | 1 | Major revision | 1 | 0.9 | 1 | 0.9 | Valid |
|  | 4a. | 0.71 | 0.7 | 0.65 | 0.66 | Major revision or Deletion | 0.86 | 0.8 | 0.85 | 0.7 | Valid |
|  | 5a. | 0.86 | 0.9 | 0.85 | 0.9 | Minor revision | 0.86 | 0.7 | 0.85 | 0.66 | Valid |
|  | 6a. | 0.86 | 0.7 | 0.85 | 0.66 | Valid | 1 | 0.9 | 1 | 0.9 | Valid |
| Child’s Psychosocial Well-Being | 7a. | 0.86 | 0.9 | 0.85 | 0.9 | Minor revision | 0.86 | 0.9 | 0.85 | 0.9 | Valid |
|  | 8a. | 0.86 | 1 | 0.85 | 1 | Valid | 1 | 0.8 | 1 | 0.9 | Valid |
|  | 9a. | 0.86 | 0.8 | 0.85 | 0.8 | Valid | 0.86 | 0.9 | 0.85 | 0.79 | Valid |
|  | 10a. | 0.86 | 0.9 | 0.85 | 0.9 | Valid | 1 | 1 | 1 | 0.9 | Valid |
| Part 2 | | | | | | | | | | | |
| Child’s illness | 1b. | 0.71 | 0.9 | 0.85 | 0.9 | Major revision | 1 | 1 | 1 | 1 | Valid |
|  | 2b. | 0.86 | 0.9 | 0.65 | 0.9 | Valid | 1 | 0.9 | 1 | 0.9 | Valid |
|  | 3b. | 0.86 | 0.9 | 0.85 | 0.9 | Minor revision | 1 | 1 | 1 | 1 | Valid |
|  | 4b. | 0.86 | 0.6 | 0.85 | 0.5 | Minor revision | 0.86 | 0.7 | 0.85 | 0.66 | Valid |
|  | 5b. | 0.86 | 0.9 | 0.85 | 0.9 | Minor revision | 1 | 0.9 | 1 | 0.9 | Valid |
|  | 6b. | 0.86 | 0.7 | 0.85 | 0.66 | Valid | 1 | 0.9 | 1 | 0.9 | Valid |
| Child’s Psychosocial Well-Being | 7b. | 0.86 | 0.8 | 0.85 | 0.8 | Valid | 1 | 0.9 | 1 | 0.9 | Valid |
|  | 8b. | 0.86 | 0.8 | 0.85 | 0.8 | Valid | 1 | 0.8 | 1 | 0.8 | Valid |
|  | 9b. | 0.86 | 0.9 | 0.85 | 0.9 | Minor revision | 1 | 0.9 | 1 | 0.9 | Valid |
|  | 10b. | 0.86 | 0.9 | 0.85 | 0.9 | Valid | 0.86 | 1 | 0.85 | 1 | Valid |
|  | 11b. | 0.86 | 0.9 | 0.85 | 0.9 | Valid | 0.86 | 1 | 0.85 | 1 | Valid |
|  | 12b. | 0.86 | 0.7 | 0.85 | 0.66 | Valid | 0.86 | 0.9 | 0.85 | 0.9 | Valid |
|  | 13b. | 0.86 | 0.9 | 0.85 | 0.9 | Valid | 0.86 | 1 | 0.85 | 1 | Valid |
|  | 14b. | 0.86 | 0.8 | 0.85 | 0.8 | Valid | 0.86 | 0.9 | 0.85 | 0.9 | Valid |
|  | 15b. | 0.71 | 0.7 | 0.65 | 0.66 | Major revision or Deletion | 0.86 | 0.9 | 0.85 | 0.9 | Valid |
| Medical staff | 16b. | 0.71 | 0.6 | 0.65 | 0.5 | Major revision or Deletion | 0.71 | 0.9 | 0.65 | 0.9 | Major revision |
|  | 17b. | 0.71 | 0.6 | 0.65 | 0.5 | Major revision or Deletion | 1 | 0.9 | 1 | 0.9 | Valid |
| Child’s Psychosocial Well-Being | 18b. | 0.71 | 0.5 | 0.65 | 0.34 | Deletion | 1 | 0.8 | 1 | 0.8 | Valid |
|  | 19b. | 0.86 | 0.7 | 0.85 | 0.66 | Valid | 1 | 1 | 1 | 1 | Valid |
|  | 20b. | 0.57 | 0.6 | 0.4 | 0.5 | Major revision or Deletion | 0.86 | 0.9 | 0.85 | 0.9 | Valid |
|  | 21b. | 0.71 | 0.5 | 0.65 | 0.34 | Deletion | 1 | 0.8 | 1 | 0.8 | Valid |
|  | 22b. | 0.86 | 0.6 | 0.85 | 0.5 | Valid | 1 | 1 | 1 | 1 | Valid |
|  | 23b. | 0.86 | 0.8 | 0.85 | 0.8 | Valid | 1 | 0.9 | 1 | 0.9 | Valid |
|  | 24b. | 0.86 | 0.6 | 0.85 | 0.5 | Valid | 1 | 0.8 | 1 | 0.8 | Valid |

**Supplementary File 1**

*Evaluation grid*

*Nickname :*

*Date :*

**Q-PHPO : Parental Hope Questionnaire in Pediatric Oncology**

**Evaluation grid**

*After taking the time to read and answer the questionnaire*

1. **Evaluation : First part of the questionnaire**

First, we’ll ask you yo evaluate the first part of the questionnaire, entitled “What you’ve been told’.

| On reading the first part of the questionnaire,  I find that… | 1  Strongly desagree | 2  Disagree | 3  Neither agree Neither disagree | 4  Agree | 5  Strongly agree | Remarks |
| --- | --- | --- | --- | --- | --- | --- |
| *It is suitable for measuring hope* | 1 | 2 | 3 | 4 | 5 |  |
| *It’s easy to fill* | 1 | 2 | 3 | 4 | 5 |  |
| *The form is relevant* | 1 | 2 | 3 | 4 | 5 |  |
| *The instructions are clear ad unambiguous* | 1 | 2 | 3 | 4 | 5 |  |
| *Filling time is acceptable* | 1 | 2 | 3 | 4 | 5 |  |

Please tick the appropriate number (1 to 5) to express your agreement with the following statements.

Always with the aim of evaluating the first part of the questionnaire.

For each of the items below, please tick the appropriate number (1 to 5) to express the extent to which you agree with the following statements.

| On reading the first part of the questionnaire,  I find that the item about the… | Is relevant in form and content | Is clear, unambiguous | Creates discomfort | | Suitable for assesing a facet of hope | | Remarks | Suggested alternative wording |
| --- | --- | --- | --- | --- | --- | --- | --- | --- |
|  | 1 = Strongly diagree  2 = Disagree  3 = Neither agree, Neither disagree  4 = Agree  5 = Strongly agree | | | | | |  |  |
| *Cure* | 1 2 3 4 5 | 1 2 3 4 5 | | 1 2 3 4 5 | | 1 2 3 4 5 |  |  |
| *Service life* | 1 2 3 4 5 | 1 2 3 4 5 | | 1 2 3 4 5 | | 1 2 3 4 5 |  |  |
| *Treatment effectiveness* | 1 2 3 4 5 | 1 2 3 4 5 | | 1 2 3 4 5 | | 1 2 3 4 5 |  |  |
| *Pain management* | 1 2 3 4 5 | 1 2 3 4 5 | | 1 2 3 4 5 | | 1 2 3 4 5 |  |  |
| *Sequels* | 1 2 3 4 5 | 1 2 3 4 5 | | 1 2 3 4 5 | | 1 2 3 4 5 |  |  |
| *Fertility* | 1 2 3 4 5 | 1 2 3 4 5 | | 1 2 3 4 5 | | 1 2 3 4 5 |  |  |
| *Normal Schooling* | 1 2 3 4 5 | 1 2 3 4 5 | | 1 2 3 4 5 | | 1 2 3 4 5 |  |  |
| *Social life* | 1 2 3 4 5 | 1 2 3 4 5 | | 1 2 3 4 5 | | 1 2 3 4 5 |  |  |
| *Physical activity* | 1 2 3 4 5 | 1 2 3 4 5 | | 1 2 3 4 5 | | 1 2 3 4 5 |  |  |
| *Games and hobbies* | 1 2 3 4 5 | 1 2 3 4 5 | | 1 2 3 4 5 | | 1 2 3 4 5 |  |  |

Additional questions:

1. Do you think other themes should be included in the scale ?

……………………………………………………………………………………………………………

……………………………………………………………………………………………………………

……………………………………………………………………………………………………………

1. Do you have anay additional comments ?

……………………………………………………………………………………………………………

……………………………………………………………………………………………………………

……………………………………………………………………………………………………………

1. **Evaluation : Second part of the questionnaire**

First. we’ll ask you yo evaluate the second part of the questionnaire.

Please tick the appropriate number (1 to 5) to express your agreement with the following statements.

| On reading the second part of the questionnaire,  I find that… | 1  Strongly desagree | 2  Disagree | 3  Neither agree Neither disagree | 4  Agree | 5  Strongly agree | Remarques |
| --- | --- | --- | --- | --- | --- | --- |
| *It is suitable for measuring hope* | 1 | 2 | 3 | 4 | 5 |  |
| *It’s easy to fill* | 1 | 2 | 3 | 4 | 5 |  |
| *The form is relevant* | 1 | 2 | 3 | 4 | 5 |  |
| *The instructions are clear ad unambiguous* | 1 | 2 | 3 | 4 | 5 |  |
| *Filling time is acceptable* | 1 | 2 | 3 | 4 | 5 |  |

Always with the aim of evaluating the second part of the questionnaire.

For each of the items below, please tick the appropriate number (1 to 5) to express the extent to which you agree with the following statements.

| On reading the second part of the questionnaire, I find that the item about the | Is relevant in form and content | Is clear, unambiguous | Creates discomfort | | Suitable for assesing a facet of hope | | Remarks | Suggested alternative wording |
| --- | --- | --- | --- | --- | --- | --- | --- | --- |
|  | 1 = Strongly diagree  2 = Disagree  3 = Neither agree, Neither disagree  4 = Agree  5 = Strongly agree | | | | | |  |  |
| *Cure* | 1 2 3 4 5 | 1 2 3 4 5 | | 1 2 3 4 5 | | 1 2 3 4 5 |  |  |
| *Long life* | 1 2 3 4 5 | 1 2 3 4 5 | | 1 2 3 4 5 | | 1 2 3 4 5 |  |  |
| *Effective treatments* | 1 2 3 4 5 | 1 2 3 4 5 | | 1 2 3 4 5 | | 1 2 3 4 5 |  |  |
| *Suffering* | 1 2 3 4 5 | 1 2 3 4 5 | | 1 2 3 4 5 | | 1 2 3 4 5 |  |  |
| *Sequels* | 1 2 3 4 5 | 1 2 3 4 5 | | 1 2 3 4 5 | | 1 2 3 4 5 |  |  |
| *Fertility* | 1 2 3 4 5 | 1 2 3 4 5 | | 1 2 3 4 5 | | 1 2 3 4 5 |  |  |
| *Happy* | 1 2 3 4 5 | 1 2 3 4 5 | | 1 2 3 4 5 | | 1 2 3 4 5 |  |  |
| *Normal life* | 1 2 3 4 5 | 1 2 3 4 5 | | 1 2 3 4 5 | | 1 2 3 4 5 |  |  |
| *Normal Schooling* | 1 2 3 4 5 | 1 2 3 4 5 | | 1 2 3 4 5 | | 1 2 3 4 5 |  |  |
| *Social life* | 1 2 3 4 5 | 1 2 3 4 5 | | 1 2 3 4 5 | | 1 2 3 4 5 |  |  |
| *Physical activity* | 1 2 3 4 5 | 1 2 3 4 5 | | 1 2 3 4 5 | | 1 2 3 4 5 |  |  |
| On reading the second part of the questionnaire, I fond that the items about the | Is relevant in form and content | Is clear. unambiguous | | Creates discomfort | | Suitable for assesing a facet of hope | Remarks | Suggested alternative wording |
|  | 1 = Strongly diagree  2 = Disagree  3 = Neither agree, Neither disagree  4 = Agree  5 = Strongly agree | | | | | |  |  |
| *Games* | 1 2 3 4 5 | 1 2 3 4 5 | | 1 2 3 4 5 | | 1 2 3 4 5 |  |  |
| *Job* | 1 2 3 4 5 | 1 2 3 4 5 | | 1 2 3 4 5 | | 1 2 3 4 5 |  |  |
| *Family* | 1 2 3 4 5 | 1 2 3 4 5 | | 1 2 3 4 5 | | 1 2 3 4 5 |  |  |
| *Doctors who know the desease* | 1 2 3 4 5 | 1 2 3 4 5 | | 1 2 3 4 5 | | 1 2 3 4 5 |  |  |
| *Competence to provide care* | 1 2 3 4 5 | 1 2 3 4 5 | | 1 2 3 4 5 | | 1 2 3 4 5 |  |  |
| *Giving love* | 1 2 3 4 5 | 1 2 3 4 5 | | 1 2 3 4 5 | | 1 2 3 4 5 |  |  |
| *Making people happy* | 1 2 3 4 5 | 1 2 3 4 5 | | 1 2 3 4 5 | | 1 2 3 4 5 |  |  |
| *Overcomming this situation* | 1 2 3 4 5 | 1 2 3 4 5 | | 1 2 3 4 5 | | 1 2 3 4 5 |  |  |
| *Meaning to this ordeal* | 1 2 3 4 5 | 1 2 3 4 5 | | 1 2 3 4 5 | | 1 2 3 4 5 |  |  |
| *Peace of mind* | 1 2 3 4 5 | 1 2 3 4 5 | | 1 2 3 4 5 | | 1 2 3 4 5 |  |  |
| *Family activities* | 1 2 3 4 5 | 1 2 3 4 5 | | 1 2 3 4 5 | | 1 2 3 4 5 |  |  |
| *Sharing special moments* | 1 2 3 4 5 | 1 2 3 4 5 | | 1 2 3 4 5 | | 1 2 3 4 5 |  |  |
| *Support from a relative* | 1 2 3 4 5 | 1 2 3 4 5 | | 1 2 3 4 5 | | 1 2 3 4 5 |  |  |

Additional questions:

1. Do you think other themes should be included in the scale ?

……………………………………………………………………………………………………………

……………………………………………………………………………………………………………

……………………………………………………………………………………………………………

1. Is there a differcne in measurement between the frist and second parts oh the questionnaire? If so, which one? If not, why not?

……………………………………………………………………………………………………………

……………………………………………………………………………………………………………

……………………………………………………………………………………………………………

1. Do you have anay additional comments ?

……………………………………………………………………………………………………………

……………………………………………………………………………………………………………

…………………………………………………………………………………………………………

**Thank you for your participation**

# Supplementary File 2

# *French version of the Questionnaire on Parental Hope in Pediatric Oncology*

# Q-EPOPé : Questionnaire d’Espoir Parental en Oncologie-pédiatrie

# Version Garçon

Les questions suivantes portent sur la situation que vous vivez avec votre enfant malade.

Certaines questions peuvent provoquer de l’inconfort et vous demander un temps de réflexion ; sachez qu’il n’y a pas de bonnes ou de mauvaises réponses, votre sentiment immédiat sera surement une bonne indication de votre éprouvé.

Dans une première partie, ci-dessous, nous allons vous demander de vous remémorer ce qui vous a été dit, récemment, par le personnel médical à propos de la santé actuelle et du futur de votre enfant.

Veuillez utiliser l’échelle ci-dessous pour exprimer jusqu’à quel point chacun des énoncés suivants est très pessimiste (1) ou très optimiste (5). Vous avez également la possibilité d’indiquer que le personnel médical ne vous a pas transmis d’informations en cochant « on ne m’a rien dit » ou que l’information ne s’applique pas à votre situation, en cochant « ne s’applique pas ».

**PREMIERE PARTIE : Ce que l’on vous a dit**

| **Dans le contexte de la maladie de votre enfant, les informations transmises par les soignant·e·s à propos de / des….sont** | **1**  **Très**  **Pessimistes** | **2**  **Plutôt pessimistes** | **3**  **Incertaines** | **4**  **Plutôt optimistes** | **5**  **Très optimistes** | **On ne m’a rien dit** | **Ne s’applique pas** |
| --- | --- | --- | --- | --- | --- | --- | --- |
| 1a. La guérison | 1 | 2 | 3 | 4 | 5 | Rien | NSP |
| 2a. L’efficacité des traitements | 1 | 2 | 3 | 4 | 5 | Rien | NSP |
| 3a. La gestion de la souffrance physique ou de l’inconfort dû à la maladie ou aux traitements (Ex : effets secondaires) | 1 | 2 | 3 | 4 | 5 | Rien | NSP |
| 4a. Séquelles à long terme psychologiques. physiques ou cognitives | 1 | 2 | 3 | 4 | 5 | Rien | NSP |
| 5a. La fertilité  (La capacité future de votre enfant à concevoir un enfant) | 1 | 2 | 3 | 4 | 5 | Rien | NSP |
| 6a. La scolarité | 1 | 2 | 3 | 4 | 5 | Rien | NSP |
| 7a. La vie sociale | 1 | 2 | 3 | 4 | 5 | Rien | NSP |
| 8a. La pratique d’une activité physique et sportive | 1 | 2 | 3 | 4 | 5 | Rien | NSP |
| 9a. Jeux et loisirs habituels | 1 | 2 | 3 | 4 | 5 | Rien | NSP |

**SECONDE PARTIE : Vous venez de répondre à des questions portant sur ce que le personnel médical vous a dit, à présent, nous aimerions comprendre, actuellement, quels sont au plus profond de vous vos espoirs, vos souhaits, vos attentes, vos ressentis.**

Veuillez utiliser l’échelle ci-dessous pour exprimer jusqu’à quel point vous êtes tout à fait en désaccord (1) ou tout à fait d’accord (5) avec chacun des énoncés suivants. Vous avez également la possibilité d’indiquer que l’item ne s’applique pas à votre situation en cochant « ne s’applique pas ».

| *Au plus profond de moi, je crois en la possibilité que mon enfant* | 1  Pas du tout d’accord | 2  Plutôt pas d’accord | 3  Moyennement d’accord | 4  Plutôt d’accord | 5  Tout à fait d’accord | Ne s’applique pas |
| --- | --- | --- | --- | --- | --- | --- |
| 1b. Guérisse | 1 | 2 | 3 | 4 | 5 | NSP |
| 2b. Ait une longue vie | 1 | 2 | 3 | 4 | 5 | NSP |
| 3b. Ait reçu ou reçoive des traitements efficaces | 1 | 2 | 3 | 4 | 5 | NSP |
| 4b. Ait moins ou plus du tout de souffrance physique ou d’inconfort dû à la maladie ou aux traitements (Ex : effets secondaires) | 1 | 2 | 3 | 4 | 5 | NSP |
| 5b. Grandisse sans séquelle psychologique, physique ou cognitive | 1 | 2 | 3 | 4 | 5 | NSP |
| 6b. Soit fertile | 1 | 2 | 3 | 4 | 5 | NSP |
| 7b. Mon enfant puisse dépasser cette situation | 1 | 2 | 3 | 4 | 5 | NSP |
| 8b. Soit heureux·se | 1 | 2 | 3 | 4 | 5 | NSP |
| 9b. Ait une vie normale | 1 | 2 | 3 | 4 | 5 | NSP |
| 10b. Suive une scolarité épanouie | 1 | 2 | 3 | 4 | 5 | NSP |
| 11b. Ait une vie sociale épanouie | 1 | 2 | 3 | 4 | 5 | NSP |
| 12b. Pratique une activité physique et sportive | 1 | 2 | 3 | 4 | 5 | NSP |
| 13b. Puisse jouer aux jeux qu’il·elle apprécie | 1 | 2 | 3 | 4 | 5 | NSP |
| 14b. Fasse le métier qu’il·elle souhaite | 1 | 2 | 3 | 4 | 5 | NSP |
| 15b. Puisse fonder sa propre famille plus tard | 1 | 2 | 3 | 4 | 5 | NSP |
| *Au plus profond de moi, je crois en la possibilité de/d’/que* | **1**  **Pas du tout d’accord** | **2**  **Plutôt pas d’accord** | **3**  **Moyennement d’accord** | **4**  **Plutôt d’accord** | **5**  **Tout à fait d’accord** | **Ne s’applique pas** |
| 16b. Pourvoir dépasser cette situation | 1 | 2 | 3 | 4 | 5 | NSP |
| 17b. De réaliser (de nouveau) des activités ensemble, en famille | 1 | 2 | 3 | 4 | 5 | NSP |
| 18b. Partager des instants privilégiés avec un ou des proches (En dehors de mon enfant) | 1 | 2 | 3 | 4 | 5 | NSP |
| 19b. Être soutenu par un ou des proches  (En dehors de mon enfant) | 1 | 2 | 3 | 4 | 5 | NSP |
| 20b. La fratrie (de l’enfant malade) puisse être épanouie | 1 | 2 | 3 | 4 | 5 | NSP |

# Supplementary File 3

# *English version of the Questionnaire on Parental Hope in Pediatric Oncology*^[[1]](#footnote-1)^

**Q-PHPO : Parental Hope Questionnaire in Pediatric Oncology**

The following questions relate to the situation you are experiencing with your ill child.

Some of the questions may make you feel uncomfortable and may require you to think for a while. There are no right or wrong answers, and your immediate feelings will probably be a good indication of how you are feeling.

In the first part below, we're going to ask you to think back to what you've been told recently by medical staff about your child's current health and future.

Please use the scale below to indicate the extent to which each of the following statements is very pessimistic (1) or very optimistic (5). You can also indicate that medical staff have not given you any information by ticking ‘I have not been told anything’ or that the information does not apply to your situation, by ticking ‘does not apply’.

**PART ONE: What you were told**

| **In the context of your child's illness. the information provided by healthcare professionals about… is:** | **1**  **Very Pessmistics** | **2**  **Pessmistics** | **3**  **Uncertains** | **4**  **Optimistics** | **5**  **Very optimistics** | **I have not been told anything** | **Not apply** |
| --- | --- | --- | --- | --- | --- | --- | --- |
| 1a. The cure | 1 | 2 | 3 | 4 | 5 | Anything | Na |
| 2a. Treatment efficacy | 1 | 2 | 3 | 4 | 5 | Anything | Na |
| 3a. Management of physical suffering or discomfort due to illness or treatment (e.g side effects) | 1 | 2 | 3 | 4 | 5 | Anything | Na |
| 4a. Long-term psychological, physical or cognitive sequelae | 1 | 2 | 3 | 4 | 5 | Anything | Na |
| 5a. Fertility | 1 | 2 | 3 | 4 | 5 | Anything | Na |
| 6a. Schooling | 1 | 2 | 3 | 4 | 5 | Anything | Na |
| 7a. Social life | 1 | 2 | 3 | 4 | 5 | Anything | Na |
| 8a. Physical activity and sports | 1 | 2 | 3 | 4 | 5 | Anything | Na |
| 9a. Games and hobbies | 1 | 2 | 3 | 4 | 5 | Anything | Na |

**SECOND PART: You've just answered questions about what the medical staff told you. Now we'd like to understand what your deepest hopes, wishes, expectations and feelings are.**

Please use the scale below to express the extent to which you strongly disagree (1) or strongly agree (5) with each of the following statements. You can also indicate that the item does not apply to your situation by ticking “does not apply”.

| *Deep down, I believe in the possibility that my child will* | 1  Strongly disagree | 2  Rather disagree | 3  Moderately agree | 4  Rather agree | 5  Strongly agree | Not apply |
| --- | --- | --- | --- | --- | --- | --- |
| 1b. be cured | 1 | 2 | 3 | 4 | 5 | Na |
| 2b have a long life | 1 | 2 | 3 | 4 | 5 | Na |
| 3b have received or will receive effective treatments | 1 | 2 | 3 | 4 | 5 | Na |
| 4b. have less or no more physical suffering or discomfort due to the disease or treatments (e.g. side effects) | 1 | 2 | 3 | 4 | 5 | Na |
| 5b. grow up without psychological, physical or cognitive sequelae | 1 | 2 | 3 | 4 | 5 | Na |
| 6b. be fertile | 1 | 2 | 3 | 4 | 5 | Na |
| 7b. My child will be able to overcome this situation | 1 | 2 | 3 | 4 | 5 | Na |
| 8b. be happy | 1 | 2 | 3 | 4 | 5 | Na |
| 9b. have a normal life | 1 | 2 | 3 | 4 | 5 | Na |
| 10b. has a fulfilling school life | 1 | 2 | 3 | 4 | 5 | Na |
| 11b. has a fulfilling social life | 1 | 2 | 3 | 4 | 5 | Na |
| 12b. participates in physical and sporting activities | 1 | 2 | 3 | 4 | 5 | Na |
| 13b. can play the games he enjoys | 1 | 2 | 3 | 4 | 5 | Na |
| 14b. do the job he wants | 1 | 2 | 3 | 4 | 5 | Na |
| 15b. be able to start a fmily of his onw in the future | 1 | 2 | 3 | 4 | 5 | Na |
| *Deep down. I believe in the possibility that* | **1**  **Strongly disagree** | **2**  **Rather disagree** | **3**  **Moderately agree** | **4**  **Rather agree** | **5**  **Strongly agree** | **Not apply** |
| 16b. onvercome this situation | 1 | 2 | 3 | 4 | 5 | Na |
| 17b. to do (again) things together as a family | 1 | 2 | 3 | 4 | 5 | Na |
| 18b. to share special moments with someone close to me (apprt from my child) | 1 | 2 | 3 | 4 | 5 | Na |
| 19b. to be suppported by someone close to me (appart form my child) | 1 | 2 | 3 | 4 | 5 | Na |
| 20b. siblings (of the sick child) can flourish | 1 | 2 | 3 | 4 | 5 | Na |

1. We present the questionnaire translated into English for review purposes and to provide an idea of the instructions and items. However, this translation is not a validated version of the questionnaire and should not be used until content and face validation are completed in an English-speaking context. [↑](#footnote-ref-1)
